# Intervention usage for the management of low back pain in a chiropractic teaching clinic

**DOI:** 10.1186/s12998-022-00412-w

**Published:** 2022-01-09

**Authors:** Ben Csiernik, Ali Smith, Joshua Plener, Anthony Tibbles, James J. Young

**Affiliations:** 1 Canadian Memorial Chiropractic College, Toronto, Canada; 2 University of Toronto, Toronto, Canada; 3 Center for Muscle and Joint Health, University of Southern Denmark, Odense, Denmark

**Keywords:** Low back pain, Guideline, Chiropractic, Treatment, Students, Musculoskeletal conditions

## Abstract

**Background:**

Despite numerous low back pain (LBP) clinical practice guidelines, published studies suggest guideline nonconcordant care is still offered. However, there is limited literature evaluating the degree to which chiropractors, particularly students, follow clinical practice guidelines when managing LBP. The aim of this study was to evaluate the frequency of use of specific interventions for LBP by students at a chiropractic teaching clinic, mapping recommended, not recommend, and without recommendation interventions based on two clinical practice guidelines.

**Methods:**

This was a retrospective chart review of patients presenting to the Canadian Memorial Chiropractic College teaching clinic with a new complaint of LBP from January to July 2019. Interventions provided under treatment plans for each patient were extracted. Interventions were classified as recommended, not recommended, or without recommendation according to two guidelines, the NICE and OPTIMa LBP guideline.

**Results:**

1000 patient files were identified with 377 files meeting the inclusion criteria. The most frequent interventions provided to patients were manipulation/mobilization (99%) and soft tissue therapy (91%). Exercise, localized percussion, and advice and/or education were included in just under half of the treatment plans. Patient files contained similar amounts of recommended (70%) and not recommended (80%) interventions according to the NICE guideline classification, with half the treatment plans including an intervention without recommendation. Under the OPTIMa acute guideline, patient files contained similar amounts of recommended and not recommended care, while more recommended care was provided than not recommended under the OPTIMa chronic guideline.

**Conclusions:**

Despite chiropractic interns providing guideline concordant care for the majority of LBP patients, interventions classified as not recommended and without recommendation are still frequently offered. This study provides a starting point to understand the treatment interventions provided by chiropractic interns. Further research should be conducted to improve our understanding of the use of LBP guideline recommended care in the chiropractic profession.

***Trial registration*:**

Open Science Framework # g74e8.

## Background

Despite increased spending in the management of low back pain (LBP) in the past decade, global levels of disability related to LBP have not improved [1]. This may in part be due to the continued use of guideline nonconcordant care [2], as more than 200 different types of treatments are offered to LBP patients [3]. Chiropractic is a health care profession that focuses on the diagnosis and treatment of musculoskeletal disorders, with LBP being the most common reason for patients to seek chiropractic care [4–7]. The most frequently provided treatments delivered by chiropractors include manual manipulation/mobilization and soft tissue therapy [4–6]. However, a wide variety of interventions are utilized by chiropractors when managing LBP [4, 6], leading to inconsistencies within professional practice.

Clinical practice guidelines (CPG) are created to help practitioners and patients select the best available treatments for the management of medical conditions [8]. CPG’s function by recommending effective interventions, while discouraging potentially ineffective interventions [8]. Although many CPGs exist for the diagnosis and management of LBP, there continues to be underuse of high-value care and overuse of low-value care [9].

It has been previously reported that up to 73% of chiropractors follow best practice recommendations [10]. However, cross-sectional data has shown that only about half of chiropractors state that CPGs significantly impact their practice [11, 12]. Similarly, low levels of guideline-concordant care have been found in evaluations of physiotherapist practice data [13, 14]. A systematic review that evaluated audits of clinical notes found the median value of patients receiving guideline recommended care for LBP to be 50%, with the median values for not recommended care and care without recommendation to be 18% and 43%, respectively [13]. A recent study published after this systematic review found that 95% of patients with LBP seeking care in United Kingdom private physiotherapy clinics received a recommended treatment, whereas 32% of patients received a treatment with recommendations against and 34% received a treatment without a recommendation [14].

Unfortunately, limited literature exists on which interventions chiropractic students utilize when managing LBP, or if these interventions follow best practice guidelines. Ensuring chiropractic students are providing guideline concordant care may serve as an important first step in ensuring the use of evidence-based care throughout their professional career. Therefore, the primary objective of this study was to evaluate the frequency of use of specific interventions for LBP by students at a chiropractic teaching clinic. The secondary objective was to estimate the proportion of treatment plans for LBP that contain interventions classfied as recommended, not recommended, and without recommendation based on two clinical practice guidelines.

## Methods

### Study design and setting

This was a retrospective review of patient data retrieved from the Canadian Memorial Chiropractic College (CMCC) teaching clinic database. The CMCC teaching clinic is composed of eight individual clinic locations, with 25 clinicians supervising over 180 students each year. Patient files with a new diagnosis of non-pathological low back pain presenting between January 1, 2019 to July 16, 2019 were retrieved. The sampling period differs from study pre-registration (OSF# g74e8) due to limited facility access caused by the COVID-19 pandemic. This study received ethical approval by the CMCC Research Ethics Board (REB# 2008B03).

### Participants

All new patients presenting with a primary complaint of LBP at first presentation to the CMCC teaching clinic were included. Patients with multimorbid complaints in addition to their primary complaint of LBP were also included. Patients under the age of 18 years or with a specific cause of LBP (spondylolisthesis, spinal stenosis, ankylosing spondylitis, structural deformity, trauma/fracture, radicular pain due to a suspected or confirmed lumbar disc herniation, sacroiliitis, osteoporosis, rheumatological conditions, fibromyalgia, osteoarthritis, malignancy, infection, and other visceral conditions) were excluded. Identified patient files that included ongoing care for LBP at CMCC were also excluded.

### Data collection

To identify eligible patient files, clinic electronic health records were searched using billing codes for a new patient visit that included treatment of the lumbar spine. The initial visit intake form and subsequent clinical visit notes for each identified patient file were reviewed for eligibility. One research team member (BC) screened all identified patient files. Secondary reviewers (AS, JP, JY) were consulted when necessary to determine if a patient file was eligible for inclusion. Patient characteristics including age, sex, and duration of pain were extracted. For duration of pain, each patient file was categorized into acute (less than or equal to 12 weeks duration) or chronic (duration greater than 12 weeks) LBP. To adequately assess the treatment plans administered, all interventions were extracted from the clinical encounter notes from the first four weeks of treatment to a maximum of three subsequent clinical visits.

### Intervention classification

Each intervention included in the treatment plan was classified according to two guidelines, the National Institute for Health and Care Excellence (NICE) LBP guideline [15] and the Ontario Protocol for Traffic Injury Management (OPTIMa) Collaboration LBP guideline [16]. Two guidelines were used to better approximate what constitutes a recommended intervention, as variability in the classification of evidence-based interventions exists between guidelines [8].

#### NICE guideline

The NICE LBP guideline was developed to inform clinicians of best practice for the assessment and management of people over the age of 16 experiencing low back pain and sciatica [15]. It was developed by a multidisciplinary group comprised of health care professionals, researchers, and members of the public [15]. The NICE guideline was selected as it is internationally recognized, as well as rated to be the highest quality guideline for LBP [17]. Interventions were categorized as recommended, not recommended, or without recommendation (Table 2). No delineation between recommendations for acute and chronic LBP is made in the NICE guideline. For the operationalization of this project and in concordance with the NICE guideline, manual therapy was classified as not recommended, unless provided in conjunction with exercise.

#### OPTIMa guideline

The OPTIMa LBP guideline was developed as one part of a series of guidelines and reviews examining the treatment of traffic related injuries [16]. The OPTIMa LBP guideline was developed through the review of current clinical practice guidelines, treatments protocols, and other programs of care, in order to identify and categorize effective non-invasive treatment interventions for acute and chronic LBP [16]. This guideline was selected as it is included and instructed as part of the curriculum at CMCC. The OPTIMa guideline provides separate recommendations for acute and chronic LBP. As such, interventions were categorized as recommended or not recommended based upon the acute or chronic LBP status of the patient (Table 3). For the purposes of this study, exercise for those with acute LBP was considered a recommended treatment in all circumstances, as the OPTIMa guideline includes the “instruction of exercise” as part of the “structured patient education” recommendation.

### Data analysis

All statistical analyses were conducted in R version 4.0.5 [18], and figures were produced using the ggplot2 package [19]. Baseline characteristics were calculated as means and proportions. The proportion of treatment plans using each intervention was calculated by dividing the frequency of use for each intervention by the total number of treatment plans. Each intervention could only be counted once per treatment plan. Interventions were classified as either recommended, not recommended, or without recommendation according to the NICE guideline, and as recommended or not recommended according to the OPTIMa guidelines (separately for patients with acute or chronic LBP). The proportion of treatment plans containing interventions within each category were calculated according to each guideline classification. Given that patients could receive multiple interventions under one treatment plan, the proportion of treatment plans that contained interventions that are recommended, not recommended, or without recommendation summed to greater than 100% (i.e. a single treatment plan could simultaneously contain interventions that were recommended, not recommended, and without recommendation).

## Results

### Patient characteristics

A total of 1000 patient files were identified by billing codes and 377 files met the inclusion criteria. The primary reason for exclusion was a returning patient who had been receiving continuous care for a complaint of LBP (480 files). Other reasons for exclusion included diagnoses of spinal stenosis (24 files), suspected disc herniations and/or radicular pain (32 files), age under 18 years (6 files), no diagnosis of LBP (10 files), and other contraindicated diagnoses (71 files). The mean age of patients was 40.5 (SD = 16.6), and 179 (47%) patients were female. Acute LBP was reported in 160 (42%) patients, while 152 (40%) had chronic LBP, and 65 (17%) patient files did not have a reported duration of symptoms.

### Intervention choices

The percentage of treatment plans containing each intervention is presented in Table 1. Spinal manipulation/mobilization and soft tissue therapy were the most frequently included interventions. Exercise, localized percussion, and advice and/or education were each included in just under half of LBP treatment plans. The use of specific modalities, with the exception of localized percussion, was found in less than 10% of treatment plans.

**Table 1:** Percentage of interventions included in treatment plans for low back pain

Intervention	Treatment plans inclusion, n (%)
Spinal manipulation/mobilization	375 (99.5%)
Soft tissue therapy	343 (91.0%)
Exercise	182 (48.3%)
Localized percussion	179 (47.5%)
Advice and/or education	173 (45.9%)
Interferential current	37 (9.8%)
Other passive modalities	13 (3.4%)
Traction	7 (1.9%)
Laser therapy	6 (1.6%)

### Guideline supported intervention use

#### NICE LBP guideline

**Fig. 1:**
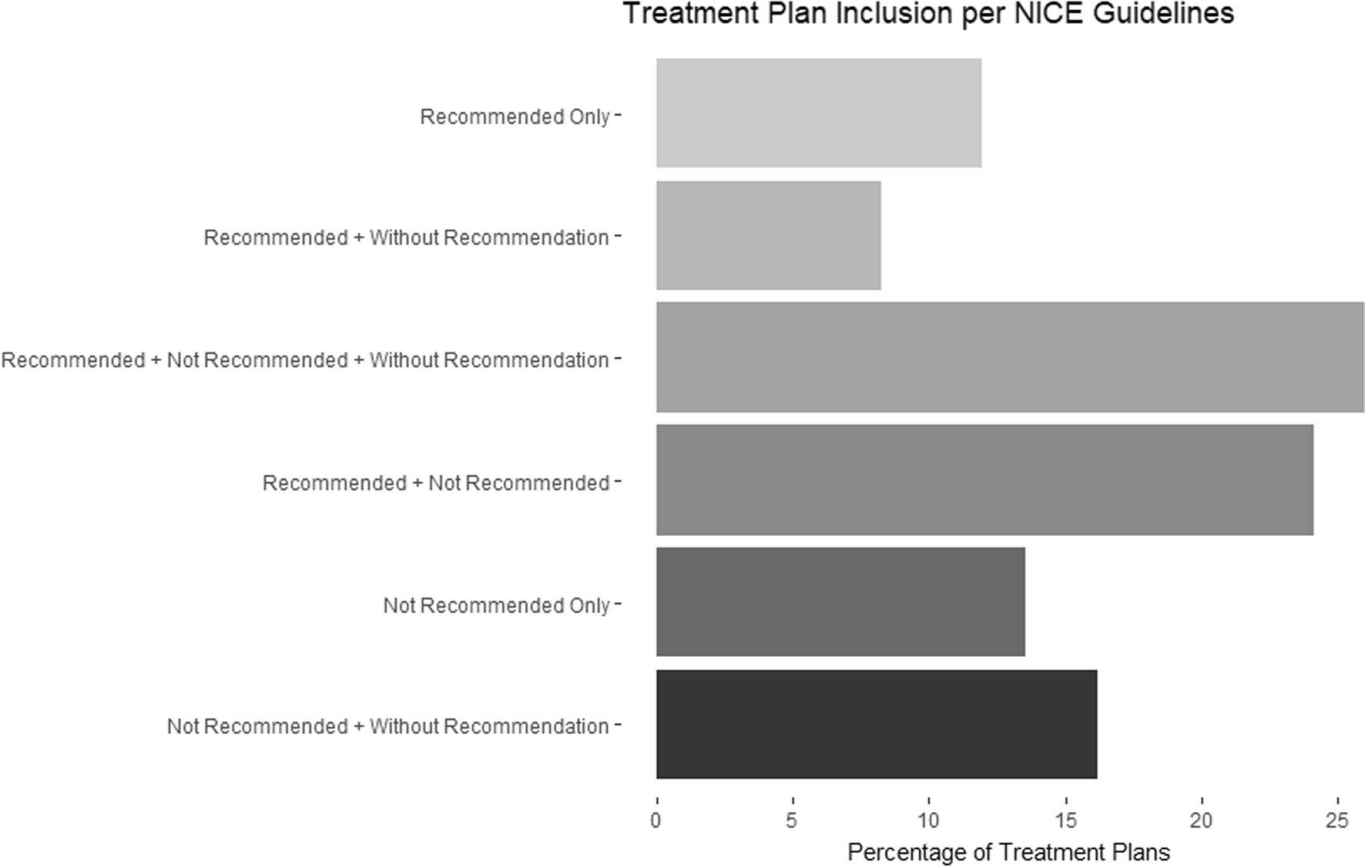
Treatment plans including combinations of recommended, not recommended, and interventions without recommendation

Interventions classified by the NICE guideline and the number of treatment plans which included interventions that were recommended, not recommended, and without recommendation are presented in Table 2. Using the NICE guideline classification, the majority of the patient files contained at least one intervention that was considered recommended (70%) and not recommended (80%). Half of the qualifying treatment plans included an intervention without recommendation. Treatment plans containing various combinations of recommended, not recommended, and interventions without recommendation are demonstrated in Fig. 1. For example, all treatment plans that contained the provision of interferential current (not recommended) and localized percussion (without recommendation) were categorized as “not recommended + without recommendation”. The most commonly provided recommended intervention was the combination of manual therapy and exercise, followed by advice and/or education. Manual therapy provided in isolation was the most common intervention utilized that was not recommended, while localized percussion was the most frequent intervention without recommendation.

**Table 2:** Intervention use and classification based on the NICE LBP Guideline

	n (%)
Total number of patient files	377 (100)
*Recommended interventions*	265 (70.3)
Education and/or advice	173 (45.9)
Manual therapy and exercise	182 (48.3)
Exercise	1 (0.3)
*Not recommended interventions*	301 (79.8)
Manual therapy alone	297 (78.8%)
Interferential current	37 (9.8%)
Traction	7 (1.9%)
Ultrasound	3 (0.8%)
Orthotics	1 (0.3%)
*Without recommendation interventions*	190 (50.4)
Localized percussion	179 (47.5%)
Laser therapy	6 (1.6%)
Other passive modalities	9 (2.4%)

The NICE guideline does not provide distinct recommendations for acute versus chronic LBP

#### OPTIMa LBP guideline

Virtually all patient files included recommended treatments for acute and chronic LBP according to the OPTIMa guideline (Table 3). The most commonly provided recommended treatments for acute LBP were spinal manipulation/mobilization, and advice and/or education. The most frequently provided treatments for acute LBP that were not recommended included soft tissue therapy, localized percussion, and interferential current. In chronic LBP, the most frequently provided recommended treatments were spinal mobilization/manipulation, and soft tissue therapy. The most frequently provided treatments that were not recommended included localized percussion and interferential current. Interventions classified by the OPTIMa guidelines are presented in Table 3.

**Table 3:** Intervention use and classification based on the OPTIMa Guidelines

	OPTIMa acute—n (%)	OPTIMa chronic—n (%)	OPTIMa unreported—n (%)
Total number of patient files	160 (42.4)	152 (40.3)	65 (17.2)
*Recommended*	159 (99.4)	152 (100)	n/a
Education and/or advice	81 (50.6)	68 (44.7)	24 (36.9)
Exercise	77 (48.1)	73 (48.0)	32 (49.2)
Spinal manipulation/mobilization	158 (98.8)	152 (100)	65 (100)
Traction	3 (1.9)	2 (1.3)	2 (3.1)
Soft tissue therapy*		133 (87.5)	59 (90.8)
*Not recommended*	158 (98.8)	80 (52.6)	n/a
Soft tissue therapy*	151 (94.4)		
Localized percussion	77 (48.1)	72 (47.4)	30 (46.2)
Interferential current	25 (15.6)	11 (7.2)	1 (1.5)
Other passive modalities	8 (5.0)	10 (6.6)	1 (1.5)

*Soft Tissue Therapy is considered as a recommended treatment for chronic LBP, but is not recommended for acute LBP. No classification could be made for the recommendation of soft tissue therapy for patient files with an unreported duration of LBP

## Discussion

The results from this study provide preliminary data on the clinician-reported management of LBP in a chiropractic teaching clinic. The most frequently recorded interventions were spinal manipulation/mobilization (99%) and soft tissue therapy (91%). Chiropractic interns provided similar amounts of interventions that were recommended and not recommended according to both the NICE guideline and the OPTIMa acute guideline. When classifying by the OPTIMa chronic guideline, interns provided more recommended interventions than not recommended interventions. The difference in results according to each guideline is notable, with the most substantial difference being that the NICE guideline does not recommend using manual therapy as a stand-alone treatment. This helps clarify our results, as over 78% of patient files reported manual therapy without exercise at one or more visits, while over 11% of all patient files reported manual therapy as the sole treatment during the recorded treatment plan.

While incongruencies between the two guidelines exist, both place an emphasis on providing education, advice, and information to patients. In this study, only 46% of patient files had records of advice and/or education being provided. However, this could have been impacted by interns’ and supervising clinicians’ beliefs that advice and/or education are not interventions in and of themselves and may not be included in patient records. Therefore, it is possible this finding is an underestimation of the true utilization of advice and/or education in the clinical management of LBP. For the operationalization of this project, we did not classify the types of advice and/or education recorded. Consequently, our results do not reflect the quality of the advice and information provided, rather, only if advice and/or education was recorded. This approach replicates the previous work by Murtagh et al. [14], though they found that physiotherapists in the United Kingdom included information and advice in 83% of patient visits. The utilization of education and advice has not been reported in previously published chiropractic practice pattern data [4, 6].

Both guidelines also place an emphasis on active rather than passive treatments [15, 16], specifically in the management of chronic LBP. In 152 patient files classified as chronic LBP, 48% of treatment plans included an exercise intervention. In the management of acute LBP, exercise was also recorded in 48% of patient files, which may indicate that the duration of a patient’s LBP may not influence chiropractic interns’ decision to include exercise during patient visits. Additionally, 83 (22%) of patient files that did not include exercise provided the combination of manual therapy and advice and/or education. We classified patient records mentioning or recommending home exercise programs as advice and/or information. Therefore, treatment plans including any form of exercise (in clinic or prescribed home exercise) may have been as high as 70% of cases. However, interventions recorded under the umbrella of advice and/or education were not only limited to home exercise prescription.

The frequency of exercise in the management of LBP by chiropractors is higher than reported in previous studies. One study showed Danish chiropractors provided exercise therapy to 38% of patients with persistent LBP of greater than six week duration [20]. In larger studies assessing chiropractors’ intervention choices across multiple conditions, the use of exercise therapy ranged from 3.5 per 100 patient visits [4] to 52 per 100 visits [6].

While this study did not assess clinical reasoning for intervention selection, a potential explanation for the low inclusion of exercise therapy could be chiropractic students’ general confidence in exercise prescription. In a survey of chiropractic students, Howitt et al. [21] found that approximately 90% of students usually or always offer counsel on exercise, and that roughly 77% of students believed exercise counselling to be highly relevant. However, previous literature has identified that between 38 and 67% of physiotherapists did not feel confident or prepared in their ability to prescribe resistance training and aerobic exercise [22], while a recent study assessing the management of persistent LBP by physiotherapy students found that passive approaches, including manual therapy, were used three times more than therapeutic exercise [23]. Furthermore, chiropractic interns may simply follow similar trends to practicing chiropractors. For example, Mior et al. [4] found that practicing chiropractors in the same Canadian province as our study used exercise in only 3.5% of patient encounters, while using manual manipulation, soft tissue therapy, and mobilisation in 72.2, 70.4 and 34.7% of patient encounters, respectfully. This may indicate that chiropractors primarily focus on hands on care, which in turn could de-emphasize the perceived value of exercise therapy. Additionally, variation in student perceptions of the identity of chiropractic may also influence the preference of passive interventions like spinal manipulation and soft tissue therapy over more active therapies [24, 25]. It is also possible that the billable services model could impact the use of various interventions by chiropractors. However, in our sample in Canada, chiropractic services are billed per appointment and not by the individual interventions provided. Therefore, this should not have impacted the results of our study.

There were limitations to this study. The collection method used to identify patient files may have led to bias in the results. To identify individual patient files matching our inclusion criteria (new patient visit, low back pain), we used billing and tracking codes associated with new patient visits, and lumbar mobilization/manipulation. This returned a specific patient file identification key, in addition to the date it was billed. Interestingly, the tracking code of lumbar mobilization/manipulation did not consistently lead to patients receiving mobilization or manipulation on the day it was billed, providing us with the best available collection method to identify new patients with a new low back complaint. The electronic health record system currently employed by CMCC does not use diagnostic coding, such as International Classification of Disease codes. Therefore, while we lack detailed description of the clinical profile of the patients included in this study, this is a pragmatic approach best suited to the affordances of the electronic records. This project was also one of the first studies attempting to extract treatment data from the CMCC electronic health record system, which broadly influenced our pragmatic approach.

Categorization of interventions, intervention descriptions, and interpretation of clinical practice guidelines do contain subjectivity and are a limitation of this study. It is possible the use of other guidelines would have led to different results for the second objective of this study [26]. We chose the NICE guidelines due to its high-quality appraisal [17], and the OPTIMa guideline was selected due to its inclusion in the CMCC curriculum and geographical considerations. Additionally, regular discussions on both interpretation and classification were conducted by the author group until agreement was reached. For example, the OPTIMa guidelines have specific timeline-based recommendations for certain interventions. We decided not to consider timeframe in the classification of interventions provided, as our definition of a treatment plan did not reach the threshold that the OPTIMa guidelines outlined (e.g. the OPTIMa guidelines recommend a maximum of nine sessions over 12 weeks when providing manipulation or mobilization to a patient with chronic LBP) [16]. Finally, as this was a retrospective chart review conducted at a chiropractic teaching clinic, there is the potential the treatment notes do not accurately reflect the actual clinical care performed. Similar issues with data interpretation have been reported when evaluating physiotherapy guideline adherence [14].

Despite the limitations of this study, our results provide insight into the care for people with LBP in a chiropractic teaching clinic. Regardless of the nuance and potential inaccuracy of the specific intervention frequency, the overall patterns of intervention use by chiropractic students warrants further discussion. The preferential use of passive therapies over active interventions does not align with best practice care recommendations for musculoskeletal disorders, where patient education, reassurance/advice, and exercise are considered fundamental tenets [27]. For example, all but two included patient files included spinal manipulation or mobilization therapy, and localized percussion therapy was used more often than education/advice and as often as exercise interventions. We can only speculate on the reasons for these observations, but it is reasonable to suspect the historical biases associated with chiropractic care may still exist in chiropractic students and educators. Educational and clinical interventions aimed at reducing the use of passive and non-recommended interventions should be developed, implemented, and tested. However, we first recommend retrospective and prospective studies similar to ours be conducted in chiropractic teaching clinics and professional practices. There are numerous chiropractic educational institutions providing direct patient care which could be used to better understand global and context-specific trends in chiropractic student management preferences.

## Conclusions

This study is the first attempt to assess the use of guideline recommended treatments in the management of LBP by chiropractic students. Most treatment plans provided spinal manipulation/mobilization and soft tissue therapy, while just under half included education/advice and exercise. Chiropractic interns provided similar amounts of interventions that were recommended and not recommended according to the NICE guideline and the OPTIMa acute guideline, while providing more recommended care according to the OPTIMa chronic guideline. This study serves as a starting point in the evaluation of treatment provided in teaching clinics, and highlights which interventions are regularly used in the management of LBP. Prospective studies assessing the management and intervention selection of LBP should be considered to gather a broad and thorough understanding of this topic.

## Data Availability

The datasets generated and/or analysed during the current study are available from the corresponding author on reasonable request.

## Abbreviations

LBP: Low back pain
CMCC: Canadian Memorial Chiropractic College
CPG: Clinical practice guideline
NICE: National Institute for Health and Care Excellence
OPTIMa: Ontario Protocol for Traffic Injury Management
